# Bony Sacral Volume after Sacro-Iliac Screw Fixation of Pelvic Fractures Is Dependent on Reduction of the Anterior Pelvic Ring

**DOI:** 10.3390/jcm12124169

**Published:** 2023-06-20

**Authors:** Florian Baumann, Stefano Pagano, Volker Alt, Viola Freigang

**Affiliations:** Department of Trauma Surgery, University Medical Centre Regensburg, Franz-Josef-Strauss-Allee 11, 93053 Regensburg, Germanyvolker.alt@ukr.de (V.A.);

**Keywords:** pelvic fracture, sacral anatomy, sacro-ilical screw fixation, volumetric measurement

## Abstract

Pelvic ring injuries are uncommon but serious injuries. Percutaneous sacro-iliac screw fixation (SSF) is the standard treatment for posterior stabilization of pelvic fractures. Compression forces of the SSF might cause deformity of the sacrum and the pelvic ring. The aim of this radio-volumetric study is to evaluate the morphometry of the sacrum and pelvic ring in SSF for posterior pelvic fractures. (1) Methods: We conducted a radio-volumetric study measuring the bony sacral volume before and after SSF for a pelvic fracture based on a three-dimensional reconstruction of the pre- and postoperative computed tomography scan of 19 patients with a C-type pelvic fracture. In addition to the bony sacral volume, we assessed the pelvic deformity and the load bearing axis. We compared the results of patients without anterior stabilization (Group A) to patients who had additional ORIF of the anterior pelvic ring. (2) Results: Median age of the patients was 41.2 years (±17.8). All patients received percutaneous SSF with partially threaded 7.3 mm screws. The sacral volume decreased from 202.9 to 194.3 cm^3^ in group A (non-operative treatment anterior, *n* = 10) and an increase of sacral volume from 229.8 to 250.4 cm^3^ in group B (anterior ORIF; *n* = 9). Evaluation of the pelvic deformity also reflected this trend by a decrease of the ipsilateral load-bearing angle in group A (37.0° to 36.4°) and an increase of this angle in group B (36.3 to 39.9°). (3) Conclusions: Bony sacral volume and pelvic deformity after sacro-iliac screw fixation in pelvic fractures depend on treatment of the anterior pelvic ring. Reduction and fixation of the anterior fracture shows an increase of the bony sacral volume and the load bearing angle leading to a closer to normal reconstruction of the pelvic anatomy.

## 1. Introduction

Pelvic ring injuries are uncommon but serious injuries. There is a significant increase in the incidence of pelvic fractures during the last two decades [1,2,3,4,5]. Although the number of high-energy pelvic fractures is decreasing, demographic changes lead to a dramatic increase in pelvic fractures of the elderly [6]. The sacral anatomy is relatively complex and shows quite some variability [7]. Therefore, evaluation of the sacral morphology with two-dimensional methods is difficult. Posterior pelvic ring injuries most frequently occur as trans-sacral fractures. The localization of the fracture is the indicator for the Denis classification, which is the most common classification for sacral fractures [8]. According to Denis, there are three different zones: trans-alar completely lateral to the neuroforamina (I), at level of the neuroforamina but not involving the spinal canal (II) or medial to the neuroforamina (III). Percutaneous sacro-iliac (SI) screw fixation is the standard treatment for un-displaced fractures of the posterior pelvic ring [2,9]. It is a minimal invasive stabilization technique providing stability by compression of the posterior pelvic ring provided by partially treaded SI-screws. A comminution zone of the ilium around the entry point of the screw is a contra-indication for SI screw fixation. Additionally, a comminution of the sacrum around the neuroforamina (Denis type II injury) is also a relative contraindication for SI screw fixation because it may lead to narrowing of the neuro-foraminal canal and compression of the sacral nerve [10]. Hyper-compression of the sacrum may lead to sacral deformity causing deformity of the posterior pelvic ring [10,11,12,13,14]. This may be a problem not only in women of childbearing age. There is no data on the three-dimensional consequences on the sacral anatomy in SI screw fixation of pelvic fractures. 

The aim of this study is to analyze the effect of SI screw fixation on sacral anatomy in pelvic ring fractures by three-dimensional monitoring of the sacral volume in a pre- and postoperative CT-based volumetry. Our hypothesis was that SI-screw fixation would lead to a decrease of the post-operative sacral volume compared to the preoperative bony sacral volume.

## 2. Methods

We prospectively recorded data of all patients sustaining a pelvic fracture in an institutional pelvic fracture register. Based on this register, we identified 19 patients with a pelvic fracture and SSF between 2017 and 2021. Inclusion criteria were:

- age over 18
- high-energy trauma
- AO/Tile C-type pelvic fracture
- sacro-iliac screw fixation (SSF)
- complete dataset regarding a preoperative and postoperative computed tomography.

Exclusion criteria were:

- age under 18
- additional stabilization procedure other than SSF
- extensive comminution zone around the sacral neuroforamen
- missing data or inadaequate image data quality of the pre- or postoperative CT.

Initial stabilization of the patients was made following the ATLS guidelines. Patients with a suspected pelvic injury received mechanical external stabilization with a pelvic binder to prevent hemorrhage [9,10,11]. All patients were diagnosed with a preoperative CT scan of the pelvis. According to Arbeitsgemeinschaft für Osteosynthesefragen (AO) guidelines, there was an indication for posterior SSF in patients with a non-displaced or minimally displaced unstable pelvic fracture involving the posterior pelvic ring. An unstable fracture of the posterior pelvic ring is a complete disruption of the posterior pelvic ring (caused by a vertical sacral fracture involving the anterior and posterior cortex of the sacrum or a separation of the anterior and posterior portion of the SI-joint). There was an indication for additional anterior open reduction and fixation if there was a higher degree of instability of the anterior pelvic ring (e.g., ligamentous injury of the symphysis or bilateral pubic ramus fracture) or a concomitant joint fracture (e.g., trans-acetabular). All patients received postoperative CT of the pelvis for evaluation of the quality of reduction and implant position.

### 2.1. Measurement Protocol

**Figure 1:**
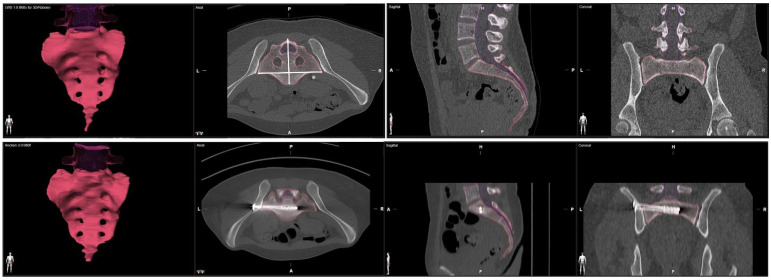
Volumetric measurement of the sacrum in preoperative and postoperative CT scans. Medio-lateral sacral diameter (MLSD) according to the Response Evaluation Criteria in Solid Tumors (RECIST) marked by *.

We processed an axial multi-planar bone kernel reconstruction (MPR) based on a preoperative and a postoperative computed tomography with a slice thickness of 1mm. We used the Brainlab Anatomical Mapping Software Package Version 1.1.1.8 of the Brainlab elements Software (Brainlab Inc., Munich, Germany) for volumetric measurement of the sacrum. The anatomical mapping software performs an automatic segmentation of the CT dataset and identifies the boundary of the sacral bone to process the volume of the sacrum. To ensure excellent data quality, we performed a manual post-processing of the automated reconstruction to adjust the exact borders of the sacral bone. We used the automatic volumetric report for calculation of the pre- and postoperative sacral volume (Figure 1). We measured the medio-lateral sacral diameter (MLSD) according to the Response Evaluation Criteria in Solid Tumors (RECIST) as the longest diameter of the sacrum within axial slices [15] (Figure 1).

For further 2D measurements two MPR were processed to calculate the pelvic center (PC) and the pelvic diameter (PD) (MPR 1) and the deviation of the pelvic center and the femoral head center (MPR 2). We used the Brainlab viewer Version 5.1.0.97 and OsiriX MD software package (Pixmeo, Bernex, Switzerland) to process these two MPRs. 

### 2.2. Measurements MPR 1

**Figure 2:**
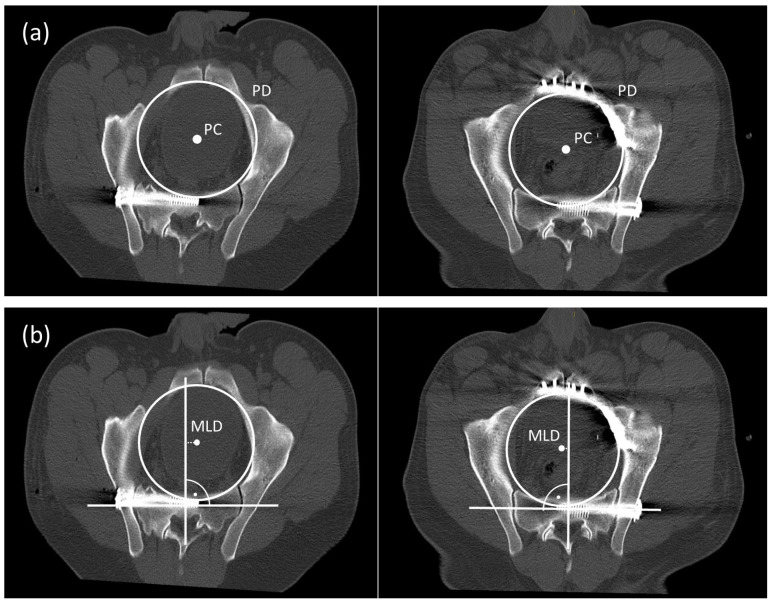
Measurement PD and PC in a CT scan without anterior stabilization (group A) and with anterior plating (group B) (**a**). Measurement of the MLD (**b**).

Figure 2 illustrates the exact methods for measurements of the anatomical deformity of the anterior and posterior pelvic ring in MPR 1. MPR1 is a reconstruction at level of the true pelvis marked by a slice cutting through the pelvic brim.

- Pelvic diameter (PD) and pelvic center (PC): We measured the diameter of the true pelvis as diameter of a circle in MPR 1 (Figure 2a). We defined the center of this circle as Pelvic Center (PC)
- Medio-lateral Deviation (MLD): The sacral axis was determined by drawing a line through the center of the first sacral body parallel to a line through both centers of the pedicle of S1. We measured the distance between the orthogonal line of the sacral axis to the PC (Figure 2b). We defined this distance as medio-lateral deviation (MLD). A positive value indicated a shift of the pelvic center towards the un-injured contralateral side, anegative value expresses a shift towards the side of the SSF.

### 2.3. Measurements MPR 2

**Figure 3:**
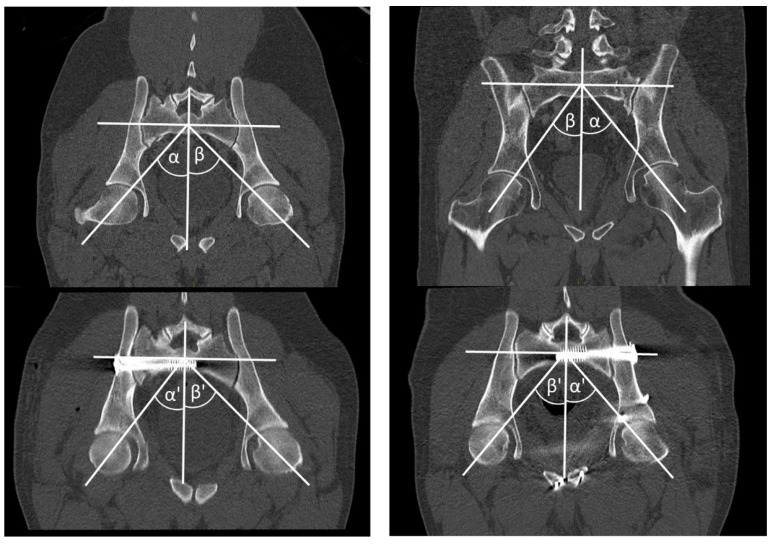
Measurement of the mechanical load-bearing axis in MPR 2. ILBA (α) and CLBA (β) in preoperative CT scan and ILBA (α’) and CLBA (β’) in postoperative CT scan.

Figure 3 illustrates the measurement of the mechanical load-bearing axis in MPR 2. MPR 2 is a reconstruction through the center of the first sacral body and through both centers of the femoral head. This reflects the biomechanical axis of the lower extremity.

- Ipsi-lateral load-bearing angle (ILBA): We measured the angle between the orthogonal to the sacral axis diameter at the center of the body S1 and the line through the center of the femoral head of the affected side.
- Contra-lateral load-bearing angle (CLBA): We measured the angle between the orthogonal to the sacral axis diameter at the center of the body S1 and the line through the center of the femoral head of the contra-lateral side.

### 2.4. Statistics

Statistical analysis was performed using the software package SPSS (Version 25, SPSS Inc., Chicago, IL, USA). Since there is no previous data this preliminary, we designed this study as an exploratory pilot study without any a priori sample size calculation. Unless otherwise stated, descriptive data are given as median ± standard deviation. For intra-individual comparison (pre OP vs post OP), we used the Wilcoxon Signed-Rank Test. We used the Chi Square Test to compare categorical data like gender or fracture classification. With the Mann-Whitney U Test, we performed non-parametric tests at group level. Level of significance was *p* < 0.05.

The Ethics Committee of the University of Regensburg approved the study in 2017 (IRB number: 17-865-104). The study was performed in accordance with the 1964 Helsinki declaration.

## 3. Results

We included 19 patients with sacro-iliac screw fixation (SSF) between 07/2017 and 04/2021 for a posterior pelvic fracture. Median age of the patients was 41.2 years (±17.8). There were 8 female and 11 male among the patients. All patients sustained a C-type pelvic ring fracture. The indication for sacro-iliac screw fixation was a non- or minimally displaced trans-iliosacral or transsacral fracture of the pelvic ring (AO OTA 61 Cx.2 or AO OTA 61 Cx.3). We classified the sacral fracture according to Denis as type I in 13 patients, type II in three patients, and type III in two patients. Two patients had an isolated ligamentous injury where the Denis classification is not applicable. All patients received percutaneous SSF with partially threaded 7.3mm screws (Synthes Inc., Zuchwil, Switzerland). In 16 patients we used two screws (12x S1, 3x S1 and S2, 1x S2), and in three patients one screw was sufficient to stabilize the posterior pelvic ring.

In 10 patients, we treated the anterior pelvic ring fracture non-operatively (Group A), the remaining 9 patients received open reduction and internal fixation (Group B) of the anterior pelvic ring (6 patients trans-pubic and three patients trans-acetabular). Table 1 gives further perioperative data. We recorded no major complication like postoperative hematoma, neurovascular injury or infection.

The median preoperative sacral volume for all patients was 226.2 cm^3^ (±39.8). For the median postoperative sacral volume, we measured 236.4 cm^3^ (±49.7). This increase of the median sacral volume was driven by an increase of the patients in group B with ORIF of the anterior pelvic ring. In group A with non-operative treatment of the anterior fracture, we recorded a postoperative reduction of the sacral volume all patients (pre OP 202.9 cm^3^ (±46.2) vs. post OP 194.3 cm^3^ (±54.4)). Table 2 reports on the three-dimensional measurement.

The pelvic diameter (PD) showed only a minimal change from 12.0 mm (±0.8) to 11.9 mm (±0.7). The decrease in PD was greater in group A (11.9 mm (±0.9) to 11.6 mm (±0.7) compared to 12.0 mm (±0.7) to 11.9 mm (±0.7) in group B), however, this was not significant (*p* = 0.638). The MLD increased in total. At group level, there was no significant difference (Table 3).

The ILBA showed a similar trend compared to the sacral volume measurement results: The ILBA decreased in group A by median −0.7° and increased by 1.5° in group B. These differences were significant (*p* = 0.014 and *p* = 0.048). The contralateral side showed no significant change measured by the CLBA (Table 4).

## 4. Discussion

The main finding of this study is that there is no decrease of the sacral volume after SFF in general. In fact, the sacral volume is dependent on reduction of the anterior pelvic fracture. We found a decrease of sacral volume in group A with SSF only and an increase in the group of patients with SSF plus ventral reduction and stabilization. The mechanical axis measured by the load-bearing angle was concordant decreasing in the SSF only group and increased in the SSF plus anterior group. Reduction and fixation of the anterior pelvic fracture led to a closer to normal reconstruction of the pelvic anatomy.

A pelvic fracture is a potentially life-threatening injury. Surgical treatment of pelvic fractures is challenging. Technical advances regarding intraoperative fluoroscopy and surgical navigation have made percutaneous sacro-iliacal screw fixation (SSF) the standard treatment for posterior stabilization of the pelvic ring [12,16,17]. However, there is ongoing discussion about posttraumatic pelvic deformity caused by sacral hyper-compression in SSF of pelvic fractures [10,18,19]. There is a number of studies investigating anterior stabilization in combined anterior and posterior pelvic fractures [9,12,16,20,21,22]. Combined stabilization is associated with longer operative time, an increased blood-loss and a longer hospital stay [12,13,23]. There is evidence that the additional anterior stabilization procedure has no negative impact on the mid-term outcome [20]. However, it is questionable if additional anterior fixation is necessary in these cases [14,22,24]. Another problem is that the study populations reported, are quite heterogenous including young patients after high-energy pelvic trauma and elderly low-energy trauma patients sustaining fragility fractures of the pelvis [1,3,5,18,19,20,22,24]. The degree of instability in these two cohorts are completely different and so are the biomechanical needs of the fixation to provide stability of the construct long enough for the fracture to heal. The different bone mineral density and the elasticity modulus in older patients might have affected the SSF-associated deformation of the pelvis. Therefore, we included only patients with a traumatic pelvic fracture after high-energy trauma and C-type instability according to the AO/Tile classification to increase generalizability of the results. The mean age of 41.2 years in our patient sample underlines this issue. There was an indication for additional anterior stabilization in patients with ligamentous instability of the anterior pelvic ring or concomitant trans-acetabular fractures requiring ORIF. This might imply a possible selection bias. However, these criteria rely solely on the anterior pelvic fracture morphology and should not affect the bony sacral volume as primary outcome measure.

Recently, Pastor et al. [19] reported on a correlation between the quality of reduction and the functional outcome in pelvic fracture patients. Since pelvic fractures are rare injuries, large studies on the functional outcome are lacking. Our study is highlighting the biomechanical consequences the treatment of the anterior pelvic ring in SSF. Although the total results showed no clear trend in change of the bony sacral volume, we found divergent results for the subgroup analysis of non-operative and surgical treatment of the anterior component in SSF for posterior pelvic fractures. This might be an important issue in planning of further studies with larger samples investigating the functional outcome. Besides functional results, specific constellations like women of childbearing potential (WOCBP) might also affect the decision-making in pelvic fractures. There is only little evidence on pelvic fractures in WOCBP [25,26,27]. However, a residual posttraumatic deformity of the pelvic ring leading to a bony narrowing of the birth channel at level of the pelvic inlet plane might lead to failure to progress during birth making it necessary to undergo cesarean section. This might be an argument for additional reconstruction of the anterior pelvic ring. However, it is unclear if the intrapelvic scaring after anterior stabilization might also be adverse for a natural childbirth. Our results indicate that a reduction of the anterior pelvic ring might prevent somewhat deformation of the posterior pelvic ring leading to a increase of bony sacral volume. However, we observed a certain amount of deformation of the pelvis ring causing asymmetry of the anterior pelvic ring in both groups quantified by the MLD and ILBA. The reconstruction of the anterior pelvic ring was closer to normal in group B, since the ipsi-lateral load-bearing angle (ILBA) increased and were found closer to the reference of the contra-lateral load-bearing angle (CLBA), whereas the ILBA in group A decreased indicating a medialization of the load bearing axis with non-operative treatment of the anterior pelvic ring. Our data do not allow a full clarification of this issue since the 3D analysis was limited to the posterior pelvic ring. We recommend further prospective 3D analyses of the anterior pelvic ring to assess the process of deformation of the anterior pelvic ring.

Assessment of the pelvic deformity is difficult [10,14,28]. **The pelvis is a complex anatomical structure and evaluation of its morphology on x-rays is largely dependent on the projection angle.** Accuracy of two-dimensional measurements for evaluation of the pelvic morphology is seen controversial [28]. We conducted all measurements in a CT-based three-dimensional dataset. We chose to track the bony sacral volume to be most accurate about a potential sacral deformity. Main feature of this study is the tracking of sacral volume in a posttraumatic condition. We expected a decrease of the bony sacral volume for all patients with SSF. The first analysis revealed conflicting results. For some patients, we recorded a decrease of volume as expected, but for others we found the sacral volume to increase. Further evaluation showed that the patients with increase of the volume had one parameter in common, that had not been in our spotlight: the reduction and stabilization of the anterior pelvic ring. The subgroup analysis revealed a connection between the dimensions of the posterior bony pelvis and the treatment of the anterior pelvic ring. We recorded a decrease of sacral volume in patients with non-operative treatment of the anterior component and a decrease of the load-bearing angle indicating an internal rotation of the hemi-pelvis of the affected side. The preoperative CT scan was conducted under pelvic compression of a pelvic binder to prevent hemorrhage. This might lead to over-compression of the bony pelvis leading to over-estimation of the preoperative pelvic deformity. Nerve root compression might be a consequence of hyper-compression of the sacrum in SSF for posterior pelvic fractures [10]. In literature, the rate for neurologic impairment after SSF is around 2.5% [12]. In our study, we did not observe any postoperative neurovascular injury neither caused by over-compression nor by mis-placement of a screw.

This study has some limitations. First, the number of patients is limited. A pelvic fracture is a rare condition and we included only high-energy trauma C-type injuries to limit confounding factors. Measurement of the two-dimensional parameters can be difficult in cases of posttraumatic deformity; however, we conducted the measurements based on CT-data to assure greatest measurement accuracy. Preclinical management of a pelvic fracture includes stabilization with a pelvic binder. As a consequence, the patients wore a pelvic binder on the preoperative CT scan. This is a potential source of bias because the binder might cause hyper-compression of the pelvis. However, this refers to both patient groups and reflects clinical reality. We compared all parameter at group level and intra-individually. Further registry-based studies are needed to confirm these findings.

## 5. Conclusions

Key feature of this study is the evaluation of the bony sacral volume in SSF for traumatic instability of the posterior pelvic ring. The main finding of this study is that the sacral volume is dependent on reduction of the anterior pelvic fracture. We found a decrease of the bony sacral volume the non-operative group and an increase of the sacral volume in patients who received additional anterior stabilization. The mechanical axis measured by the load-bearing angle was concordant decreasing in the SSF only group and increased in the SSF plus anterior group. Reduction and fixation of the anterior pelvic fracture led to a closer to normal reconstruction of the pelvic anatomy.

## Figures and Tables

**Table 1:** Demographic and perioperative data.

*n* = 19	Group A (*n* = 10)		Group B (*n* = 9)		Total		*p*
Age	40.3 (±12.6)		42.7 (±20.6)		41.2 (±17.8)		0.873
Gender	6 female/4 m		2 f/7 m		8 f/11 m		0.170
Pelvic Fracture Classification (AO OTA)	6x C1.2, 3x C1.3, 1x C3.3		4x C1.2, 4x C1.3, 1x C2.3		10x C1.2, 7x C1.3, 1x C2.3, 1x C3.3		0.782
Sacral Fracture Classification (Denis)	I	8	I	5	I	13	0.841
	II	1	II	2	II	3	
	III	0	III	1	III	1	
	Not applicable	1	Not applicable	1	Not applicable	2	
No. of Screws	1 only 1 screw		2 only 1 screw		3 only 1 screw		0.466

**Table 2:** Three-dimensional pre- and postoperative sacral volume measurement.

*n* = 19	Group A	Group B	Total	*p*
Median pre OP Sacral Volume (in cm^3^)	202.9 cm^3^ (±46.2)	229.8 cm^3^ (±31.7)	226.2 cm^3^ (±39.8)	0.453 (A vs. B)
Median post OP Sacral Volume (in cm^3^)	194.3 cm^3^ (±54.4)	250.4 cm^3^ (±37.8)	236.4 cm^3^ (±49.7)	0.757 (A vs. B)
*p*	0.014 *	0.041 *	0.857	

* Significant at *p* < 0.05.

**Table 3:** Medio-lateral deviation (MLD) measurement results (in MPR 1).

*n* = 19	Group A	Group B	Total	*p*
Median pre MLD (in mm)	−0.3 mm (±4.7)	1.4 mm (±4.6)	0.3 mm (±4.4)	0.928 (A vs. B)
Median post OP MLD (in mm)	4.3 mm (±5.6)	4.7 mm (±3.1)	4.6 mm (±4.4)	0.303 (A vs. B)
*p*	0.276	0.784	0.041 *	

* Significant at *p* < 0.05.

**Table 4:** Ipsilateral load-bearing angle (ILBA) measurement results (in MPR 2).

*n* = 19	Group A	Group B	Total	*p*
Median pre ILBA α (in degree)	37.0° (±3.5)	36.4° (±3.4)	36.9° (±3.5)	0.749 (A vs. B)
Median post OP ILBA α’ (in degree)	36.3° (±2.6)	39.9° (±5.5)	37.3° (±4.6)	0.116 (A vs. B)
*p*	0.014 *	0.048 *	0.849	
Median pre CLBA β (in degree)	40.2° (±3.7)	41.2° (±4.1)	40.8° (±3.8)	0.610 (A vs. B)
Median post OP CLBA β’ (in degree)	41.2° (±2.0)	42.4° (±5.3)	40.8° (±3.9)	0.889 (A vs. B)
*p*	0.784	0.757	0.294	

* Significant at *p* < 0.05.

## Data Availability

Not applicable.
